# Depressive symptoms and mortality‐findings from Helsinki birth cohort study

**DOI:** 10.1111/acps.13512

**Published:** 2022-11-01

**Authors:** Mia D. Eriksson, Johan G. Eriksson, Päivi Korhonen, Hannu Koponen, Minna K. Salonen, Tuija M. Mikkola, Eero Kajantie, Niko S. Wasenius, Mikaela von Bonsdorff, Hannu Kautiainen, Merja K. Laine

**Affiliations:** ^1^ Department of General Practice and Primary Health Care University of Helsinki and Helsinki University Hospital Helsinki Finland; ^2^ Folkhälsan Research Center Helsinki Finland; ^3^ Doctoral Programme of Population Health, Faculty of Medicine University of Helsinki Helsinki Finland; ^4^ Department of Obstetrics & Gynecology and Human Potential Translational Research programme, Yong Loo Lin School of Medicine National University of Singapore Singapore Singapore; ^5^ Singapore Institute for Clinical Sciences (SICS) Agency for Science, Technology and Research (A*STAR) Singapore Singapore; ^6^ Turku University Hospital and University of Turku Turku Finland; ^7^ Department of Psychiatry University of Helsinki and Helsinki University Hospital Helsinki Finland; ^8^ Department of Public Health Solutions, Public Health Promotion Unit Finnish Institute for Health and Welfare Helsinki Finland; ^9^ Clinicum, Faculty of Medicine University of Helsinki Helsinki Finland; ^10^ PEDEGO Research Unit, MRC Oulu Oulu University Hospital and University of Oulu Oulu Finland; ^11^ Department of Clinical and Molecular Medicine Norwegian University of Science and Technology Trondheim Norway; ^12^ Gerontology Research Center and Faculty of Sport and Health Sciences University of Jyväskylä Jyväskylä Finland; ^13^ Institute of Public Health and Clinical Nutrition University of Eastern Finland Kuopio Finland

**Keywords:** cohort studies, comorbidity, depression, depressive disorder, mortality

## Abstract

**Background:**

Individuals with depression and depressive symptoms have a higher mortality rate than non‐depressed individuals. The increased comorbidity and mortality associated with depression has remained largely unexplained. The underlying pathophysiological differences between depressive subtypes, melancholic and non‐melancholic, may provide some explanation to this phenomenon.

**Methods:**

One thousand nine hundred and ninety five participants (mean age 61 years) from the Helsinki Birth Cohort Study were recruited for this prospective study and followed up for a mean of 14.1 years. Information regarding medical history, lifestyle, and biochemical parameters were obtained. Depressive symptoms were assessed using the Beck Depression Inventory. Standardized mortality ratios were calculated.

**Results:**

Participants were followed up for a total of 28,044 person‐years. The melancholic depressive group had an increased adjusted risk of mortality [HR 1.49 (95% CI: 1.02–2.20)] when compared to the non‐depressive group. Comparing mortality to the whole population of Finland using standardized mortality ratios (SMR) both the non‐melancholic [1.11 (95% CI: 0.85–1.44)] and melancholic depressive [1.26 (95% CI: 0.87–1.81)] groups had higher mortality than the non‐depressive group [0.82 (95% CI: 0.73–0.93)].

**Conclusions:**

Melancholic depressive symptoms are most strongly related to a higher mortality risk.

## Highlights

- Depression is associated with mortality.
- Depressive symptoms based on BDI‐IA show an association with increased risk for mortality.
- Specifically melancholic depressive symptoms present with increased risk for mortality.

**Significant outcomes**

- Depressive symptoms, and specifically melancholic depressive symptoms, are associated with an increased risk for mortality. There was, however, no differences between causes of death between groups.

**Limitations**

- The lack of clinical interviews to categorize participants into depressive subtype groups, and utilization of only questionnaires is a limiting factor. The study sample, being from a birth cohort, is homogenous and may therefore limit the applicability of findings to other groups of people.

## 1 MORTALITY AND DEPRESSIVE SYMPTOMS‐FINDINGS FROM HELSINKI BIRTH COHORT STUDY

Depression affects over 322 million people worldwide, making it the leading cause of disability in the world^1^ and an enormous contributor to the overall burden of disease.^2^ Depression affects about 7% of the older population, and accounts for 5.7% of years lost to disability in the population above the age of 60.^3^ The World Health Organization has predicted depression to be the leading cause of burden of disease by the year of 2030.^4^

Depression is associated with a higher rate of all‐cause mortality,^5^, ^6^, ^7^, ^8^, ^9^ as well as cardiovascular disease (CVD)^6^, ^8^ and cancer mortality.^8^ Neither socioeconomic factors nor health behaviors can fully account for this difference.^8^, ^10^ Some have even suggested that a current depressive episode should be considered life‐threatening.^11^

Depression can be categorized into two subtypes: melancholic and non‐melancholic.^12^, ^13^ It has been indicated that melancholic and non‐melancholic depression may be associated with different pathophysiology,^12^ which could be an explanation for differences in mortality. Dysregulation of inflammatory and metabolic pathways have been associated with non‐melancholic depression.^12^, ^13^ On the other hand, hypothalamic–pituitary–adrenal (HPA) axis dysregulation has been associated with melancholic depression.^12^, ^13^ Some suggest HPA‐axis overactivity to be related to melancholic depression and underactivity to non‐melancholic depression.^14^ These differences attempt to explain parts of the pathophysiology of depression and its subtypes,^13^ but much still remains unknown.

Previous studies have mostly focused on mortality among the depressed as one group, ignoring the heterogeneity and subdivision of depressive groups. The aim of this study was to assess the differences in mortality between melancholic and non‐melancholic depression. We hypothesized that differences in mortality could at least partly be explained by subtype of depression.

## 2 METHODS

### 2.1 Subjects

The Helsinki Birth Cohort Study (HBCS) consists of 13,345 men and women born 1934–1944 in Helsinki, Finland at either the Helsinki University Hospital or Helsinki City Maternity Hospital. All participants attended child welfare clinics in Helsinki. The majority also attended school in Helsinki. Records regarding birth, child welfare, and school health have been detailed in prior publications.^15^, ^16^

By 1971 a unique identification number was assigned to each member of the Finnish population. With these identification numbers 8760 individuals (4630 men and 4130 women) born at Helsinki University Hospital were identified from the original cohort. Participants from this pool who were alive and living in Finland were selected for a clinical study in 2001–2004 using random‐number tables. Of the 2902 participants identified and invited, 2003 took part in the study. Eight participants were excluded due to missing data, resulting in the total number of participants for the study at hand to be 1995. The mortality follow‐up ended December 31, 2018.

### 2.2 Ethics

The Coordinating Ethical Committee of the Hospital District of Helsinki and Uusimaa approved the study protocol. Each subject gave written informed consent prior to participation in any part of the study. Ethics outlined by the declaration of Helsinki were followed throughout all study procedures.

### 2.3 Depressive symptoms

To assess depressive symptoms among subjects the Beck Depression Inventory (BDI) was used. The BDI is a 21‐category depression questionnaire of self‐reported behavioral manifestations of specific attitudes and symptoms.^17^ The scores range between a total of 0 and 63 points, with a score ≥10 total points having been validated to screen for subjects with clinical depression.^18^ The ≥10 point cut‐off is designed to screen for even mild depression, but identifying those scoring <10 points as not showing significant enough symptoms to fit a depression diagnosis.^18^

Participants scoring ≥10 points were divided into a melancholic depressive (BDI questions 1, 4, 7–12, 16, 18–19) and a non‐melancholic (BDI questions 2–3, 5–6, 13–15, 17, 20–21) depressive group based on the greater mean score of symptoms according to the Diagnostic and Statistical Manual of Mental Disorders IV (DSM‐IV). The melancholic symptoms used were sadness, past failure, loss of pleasure, guilty feelings, punishment feelings, loss of interest, irritability, change of sleeping and appetite. This division has been previously described.^19^, ^20^, ^21^, ^22^ The DSM‐IV specifies eight criteria for the melancholic specifier of mood disorders, A.1–2 and B.1–6. The BDI questions on sadness and loss of interest satisfy the DSM‐IV melancholic features criteria A.1, and the BDI question on loss of pleasure satisfies criteria A.2. Criteria B.1 is represented by the BDI question on irritability. DSM criteria B.3, B.5, and B.6 are represented by the BDI questions regarding changes in sleep, appetite and weight changes, and punishment feelings, respectively. The criteria B.2 (diurnal variation) and B.4 (psychomotor symptoms), have not been assessed.

### 2.4 Other measurements

Blood samples from each participant were drawn for laboratory assessments after an overnight fast. A hexokinase method was used for evaluation of plasma glucose at 0 min (fasting) and 120 min after a 75 g oral glucose tolerance test (OGTT). Standard enzymatic methods were utilized for the evaluation of serum total cholesterol and triglycerides.^23^, ^24^

The height and weight of each participant were measured using a Kawi stadiometer and Seca Alpha 770 scale, respectively. Body mass index (BMI) was calculated as weight (kg) divided by height (m^2^). Blood pressure was measured with a standard sphygmomanometer from the participant's right arm while seated. The reported measurement was the mean of two successive readings.

A validated Kuopio Ischaemic Heart Disease Risk Factor Study 12‐month history questionnaire was used to assess leisure‐time physical activity (LTPA).^25^ Participants were asked about their physical activity, frequency, duration, and intensity over the previous year. Each activity was assigned a metabolic equivalent of task (MET)‐value based on database information (1 MET = 3.5 ml O_2_/kg/min).^26^ MET values for each activity were multiplied with average duration and frequency per week and then summed up to calculate the total LTPA (METh/week).

Questionnaires were used to gather self‐reported information on health status, smoking habits, alcohol consumption and socioeconomic variables (economic status, years of education, and cohabitation).

### 2.5 Statistical analysis

Data are presented as means with standard deviation (SD) or as counts (*n*) with percentages (%). Statistical comparisons between depressive groups were performed using analysis of variance (ANOVA), a chi‐square test, or generalized linear model. Post hoc comparisons of group pairs were performed by Hommell's multiple comparison test after statistical comparison had established significant differences among the groups. The Kaplan–Meier method was used to estimate the cumulative all‐cause mortality. Adjusted Kaplan–Meier cumulative mortality rates were estimated using inverse probability weighting (IPW); 95% confidence intervals were obtained by bias corrected bootstrapping (5000 replications). Adjustments were made for age, sex, educational attainment, smoking status, diabetes mellitus, systolic blood pressure, cardiovascular disease, body mass index, and dyslipidemia. Cox proportional hazard regression was used to estimate the hazard ratios (HR) and their 95% confidence intervals (CI). The possible non‐linear relationship between BDI and all‐cause mortality was modeled using restricted cubic splines with four knots at the 5th, 35th, 65th, and 95th percentiles; knot locations are based on Harrell's recommended percentiles.^27^ The proportional‐hazards assumption was evaluated by Schoenfeld residuals and log–log plots. In the case of violation of the assumptions (e.g., non‐normality) for continuous variables, a bootstrap‐type method was used, and Monte–Carlo *p*‐values were calculated for categorical variables when the number of observations were small. The ratio of observed to expected number of deaths, the standardized mortality ratio (SMR) for all‐cause deaths, was calculated using subject‐year methods with 95% CI. The expected number of deaths was calculated based on sex‐, age‐ and calendar‐period‐specific mortality rates in the Finnish population (Official Statistics of Finland). The normality of variables was evaluated graphically and using the Shapiro–Wilk test. All analyses were performed using STATA software, version 17.0 (StataCorp LP, College Station, TX).

## 3 RESULTS

**FIGURE 1:**
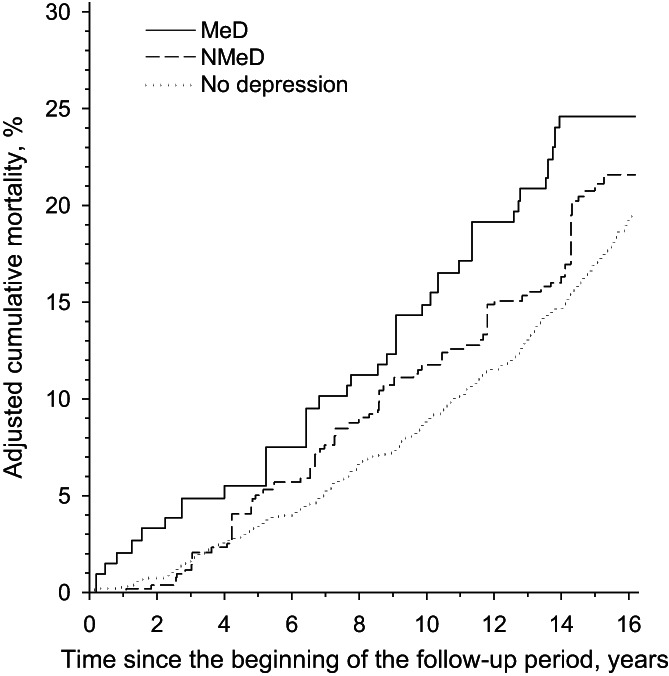
All‐cause mortality over time according to depressive symptoms. Adjusted for diabetes mellitus, age, sex, educational attainment, systolic blood pressure, smoking status, cardiovascular disease, body mass index, and dyslipidemia. MeD, melancholic depressive group; NMeD, non‐melancholic depressive group, No depression = Beck Depression Inventory score < 10. Adjusted 15‐year mortality rates were 17.0% (95% CI: 18.9–15.3) for the non‐depressive group, 21.1% (95% CI: 28.1–15.2) for the non‐melancholic depressive group, and 24.6% (95% CI: 33.6–16.5) for the melancholic depressive group

The mean follow‐up time was 14.1 years. The cohort as a whole was followed‐up for a total of 28,044 person‐years, of which 22,677 were in the non‐depressive group, 3773 in the non‐melancholic depressive group, and 1594 were in the melancholic depressive group. During the follow‐up a total of 357 participants died. Of these 273 were in the non‐depressive group, 55 in the non‐melancholic depressive group, and 29 in the melancholic depressive group. The crude mortality rate throughout the follow‐up period was 19.2% (95% CI: 17.0–21.6) for the non‐depressive group, 21.7% (95% CI: 16.9–27.5) for the non‐melancholic depressive group, and 24.4% (95% CI: 17.7–33.2) for the melancholic depressive group. The p‐value for the crude difference between the three groups was 0.051. After adjusting the mortality rate of each group for diabetes mellitus, age, sex, educational attainment, systolic blood pressure, smoking status, cardiovascular disease, BMI, and dyslipidemia there was still no difference when comparing the three groups (*p* = 0.11). Figure 1 shows that the adjusted cumulative mortality does not differ between the non‐depressive and non‐melancholic depressive groups.

Table 1 describes the characteristics of all participants per group. There are more women in the non‐melancholic group than in either of the other two groups. The non‐melancholic group is less educated and less likely to be cohabitating or consuming alcohol than the non‐depressive group. They, however, are more likely to be current smokers. The non‐melancholic group has higher mean BMI than either of the other groups, and higher mean glucose concentration at 120 min post OGTT than the non‐depressive group. The non‐melancholic group also has more diabetes, pulmonary disease, and cardiovascular disease than the non‐depressive group. The melancholic group has both lower systolic and diastolic blood pressure, total cholesterol concentrations, and LDL‐cholesterol concentrations than either of the other two groups. There is no difference between the mean BDI scores for the non‐melancholic depressive group and the melancholic depressive group (*p* = 0.62).

**TABLE 1:** Characteristics of participants (*N* = 1995)

	BDI score < 10 (0) *n* = 1603 A	NMeD *n* = 273 B	MeD *n* = 119 C	*p* Value [multiple comparison]^a^
Women, *n* (%)	806 (50)	200 (73)	61 (51)	<0.001 [A/B, B/C]
Age (years), mean (SD)	61 (3)	62 (3)	61 (3)	0.052
Education (years), mean (SD)	12.4 (3.7)	11.5 (3.4)	12.1 (3.6)	0.002 [A/B]
Cohabitating, *n* (%)	1261 (79)	187 (68)	85 (71)	<0.001 [A/B]
Current smokers, *n* (%)	372 (23)	81 (30)	33 (28)	0.048 [A/B]
Alcohol consumption ≥1/week, *n* (%)	849 (53)	114 (42)	53 (45)	0.001 [A/B]
LTPA (METh/week), mean (SD)	38 (27)	36 (30)	39 (27)	0.91
BMI (kg/m^2^), mean (SD)	27.4 (4.3)	29.2 (6.2)	27.6 (4.9)	<0.001 [A/B, B/C]
BP (mmHg), mean (SD)				
Systolic	146 (20)	147 (19)	139 (19)	<0.001 [A/C, B/C]
Diastolic	89 (10)	89 (11)	85 (9)	<0.001 [A/C, B/C]
Glucose (mmol/l), mean (SD)				
0 min (fasting)	5.81 (1.24)	5.98 (1.65)	6.15 (1.97)	0.009 [A/C]
120 min (post OGTT)	7.74 (3.28)	8.53 (3.92)	8.33 (4.09)	0.001 [A/B]
Total cholesterol (mmol/l), mean (SD)	5.95 (1.06)	5.99 (1.09)	5.71 (1.26)	0.049 [A/C, B/C]
LDL‐cholesterol (mmol/l), mean (SD)	3.68 (0.89)	3.65 (0.92)	3.40 (0.82)	0.006 [A/C, B/C]
HDL‐cholesterol (mmol/l), mean (SD)	1.60 (0.43)	1.62 (0.44)	1.56 (0.48)	0.43
Triglycerides, (mmol/l), mean (SD)	1.48 (0.78)	1.63 (0.92)	1.80 (2.91)	0.001 [A/C]
Diseases, *n* (%)				
CVD	643 (40)	141 (52)	55 (46)	0.001 [A/B]
Pulmonary	142 (9)	50 (18)	17 (14)	<0.001 [A/B]
Cancer	109 (7)	26 (10)	6 (5)	0.18
DM	235 (15)	58 (21)	21 (18)	0.019 [A/B]
BDI, mean (SD)	3.8 (2.7)	14.3 (5.1)	14.1 (5.2)	

*Note*: All diseases are self‐reported by the participants. cvd diseases: hypertension, angina pectoris, intracardiac thrombi, stroke, heart failure, arrhythmias. Pulmonary diseases: asthma, COPD.

Abbreviations: BDI, Beck Depression Inventory; BMI, body mass index; BP, blood pressure; CVD, cardiovascular disease; DM, diabetes mellitus; HDL, high density lipoprotein cholesterol; LDL, low density lipoprotein cholesterol; LTPA, leisure time physical activity; OGTT, oral glucose tolerance test; SD, standard deviation.

^a^ Hommel's pairwise multiple comparison procedure was used to correct significance levels for post hoc testing (*p* < 0.05).

**FIGURE 2:**
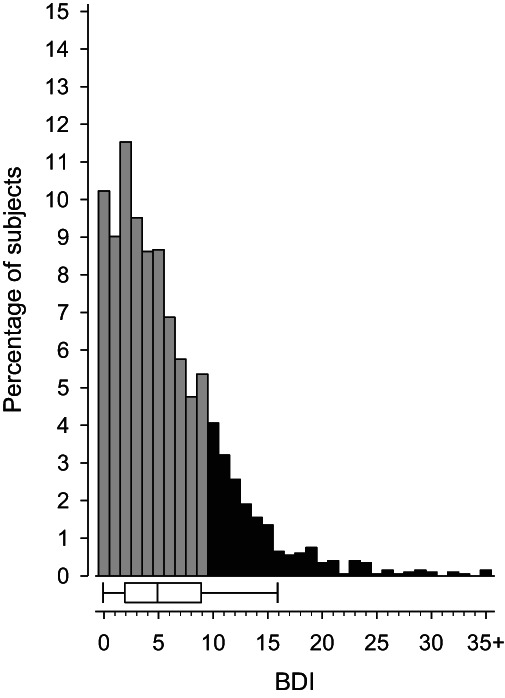
Distribution of Beck Depression Inventory (BDI) scores in the study population. Boxplot shows median and interquartile range. Whiskers indicate 5th and 95th percentiles. The black represents those scoring ≥10 on the BDI.

**FIGURE 3:**
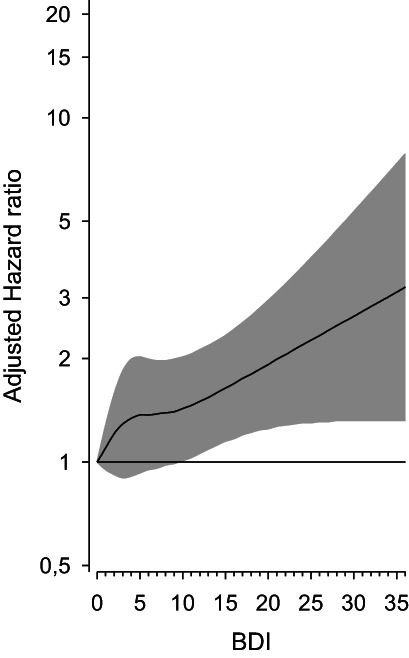
Hazard Ratio for all‐cause mortality in relation to BDI score. Adjusted for age, sex, educational attainment, smoking status, diabetes mellitus, systolic blood pressure, cardiovascular disease, body mass index, and dyslipidemia. BDI = Beck Depression Inventory. The gray area represents the 95% confidence interval. A BDI score of zero is set as the reference point (HR 1.00)

Figure 2 shows the distribution of BDI scores among the participants. The hazard ratio for mortality increases with increasing BDI scores (Figure 3). A BDI score above 10 has a hazard ratio that significantly differs from a score of zero.

Hazard ratios using mortality of the non‐depressive group as the comparison (Table 2) show that there is no increased mortality risk for the non‐melancholic group in the crude model or either of the adjusted models. There is a higher risk of mortality in the melancholic depressive group in the crude model (HR 1.53, 95% CI: 1.04–2.42). In the fully adjusted model (adjusted for diabetes mellitus, age, sex, educational attainment, systolic blood pressure, smoking status, cardiovascular disease, BMI, and dyslipidemia) the melancholic depressive group still has a higher mortality risk (HR 1.49, 95% CI: 1.02–2.20) than the non‐depressive group.

**TABLE 2:** Hazard ratio (HR) of mortality per depressive group in comparison to the non‐depressive group (BDI <10)

	Model I	Model II	Model III
	HR (95% CI)	HR (95% CI)	HR (95% CI)
BDI <10	1.00 (Reference)	1.00 (Reference)	1.00 (Reference)
NMeD	1.22 (0.91–1.63)	1.25 (0.93–1.69)	1.12 (0.83–1.52)
MeD	1.53 (1.04–2.42)	1.43 (0.98–2.10)	1.49 (1.02–2.20)

*Note*: Model I: crude. Model II: adjusted for age, sex, education, and smoking status. Model III: Adjusted for age, sex, educational attainment, smoking status, diabetes mellitus, systolic blood pressure, cardiovascular disease, body mass index, and dyslipidemia.

Abbreviations: BDI, Beck Depression Inventory; BDI <10, non‐depressive group; NMeD, non‐melancholic depressive symptoms; MeD, melancholic depressive symptoms.

Causes of death have been detailed in Table 3. Neoplasms represent the highest percentage of causes of death for each group individually, as well as overall. The situation is the same for the second most common cause of death, diseases of the circulatory system.

**TABLE 3:** Causes of death by ICD‐10 code per depressive group

Cause of Death	BDI score < 10 *n* = 273	NMeD *n* = 55	MeD *n* = 29
Certain infectious and parasitic diseases, A00‐B99, *n* (%)	1 (0.4%)	1 (1.8%)	0 (0.0%)
Neoplasms, C00‐D48, *n* (%)	134 (49.1%)	21 (38.2%)	12 (41.4%)
Diseases of the blood and blood‐forming organs and certain disorders involving the immune mechanism, D50‐D89, *n* (%)	1 (0.4%)	0 (0.0%)	0 (0.0%)
Endocrine, nutritional and metabolic diseases, E00‐E90, *n* (%)	2 (0.7%)	0 (0.0%)	0 (0.0%)
Mental, Behavioral and Neurodevelopmental disorders, F00‐F99, *n* (%)	2 (0.7%)	1 (1.8%)	0 (0.0%)
Diseases of the nervous system, G00‐G99, *n* (%)	25 (9.2%)	4 (7.3%)	3 (10.3%)
Diseases of the circulatory system, I00‐I99, *n* (%)	70 (25.6%)	19 (34.6%)	9 (31.0%)
Diseases of the respiratory system, J00‐J99, *n* (%)	12 (4.4%)	3 (5.5%)	3 (10.3%)
Diseases of the digestive system, K00‐K93, *n* (%)	9 (3.3%)	3 (5.5%)	1 (3.5%)
Symptoms, signs and abnormal clinical and laboratory findings, not elsewhere classified, R00‐R99, *n* (%)	3 (1.1%)	0 (0.0%)	0 (0.0%)
External causes of morbidity, V01‐Y98, *n* (%)	14 (5.1%)	3 (5.5%)	1 (3.5%)

Abbreviations: ICD, international classification of diseases; BDI, Beck Depression Index; BDI score < 10, non‐depressive group; NMeD, non‐melancholic depressive group; MeD, melancholic depressive group.

When the mortality of the study population was compared to that of the general population (SMR), we found that the standardized mortality ratio for the non‐depressive group [0.82 (95% CI: 0.73–0.93)] was lower than that of the general population, while no difference was detected for melancholic [1.26 (0.87–1.81)] or non‐melancholic [1.11 (0.85–1.44)] depressive groups. However, mortality ratios were higher in the non‐melancholic depressive group (*p* = 0.044) and in the melancholic depressive group (*p* = 0.031) than in the non‐depressive group.

## 4 DISCUSSION

We showed that those participants with a BDI score in the depressive range had a higher mortality risk than those participants scoring in the non‐depressive range, which was also reflected in the population comparison. The fact that we found no differences between the BDI scores of the melancholic and the non‐melancholic depressive groups supports the theory that it may not be the severity of depression but rather the type of depression that differentiates the mortality of the depressive groups. In accordance with our hypothesis, we found that melancholic depression had a higher mortality risk both overall and cumulatively, when compared to the non‐depressive group. Even though we were unable to show a statistical difference between the non‐melancholic group and either of the other two groups the importance of this study lies in showing that the melancholic depressive group rather than all depressive participants have a higher mortality risk than the non‐depressive group. Another study in Finland had the opposite finding where non‐melancholic depression but not melancholic depression was associated with a higher risk of mortality.^28^ However, the participants were all part of a CVD risk group,^28^ which we believe may have influenced the mortality rates as well as the overall findings. Since our participants are a random selection from the whole population of those born in Helsinki, we believe that our study participants may better represent the general population of the country.

The most common causes of death in our cohort were neoplasms and cardiovascular causes in both depressive and non‐depressive participants. Previous research has also reported that causes of death do not differ between depressed and non‐depressed individuals.^29^ Furthermore, suicide does not explain the excess mortality among depressed individuals.^29^ Depression has however been shown to have an association with higher all‐cause mortality,^5^, ^6^, ^7^, ^8^, ^9^ CVD mortality^6^, ^8^ and cancer mortality.^8^ This shows that even though the mortality risks may differ the distribution of causes of death remains the same no matter the depressive profile.

In this study we used depressive symptoms screened for by the BDI questionnaire rather than clinically diagnosed depression. Studies have shown that clinical depression and subclinical depressive symptoms are both associated with higher mortality.^5^, ^6^, ^30^ Other research has indicated that diagnosed clinical depression has a similar mortality risk as screened depressive symptoms without official diagnosis.^31^ Based on this information, our results can be used to further the overall understanding of depression even though the current study utilized self‐reported symptomatic grouping. It would, however, have been a strength to have the depressive subgrouping done based on clinical assessments rather than BDI. The BDI has not originally been designed for subtyping depressive symptoms and may therefore not be the best possible way to find said subtypes. However, no absolute measure for categorizing depressive subtypes exists. Clinical interviews by professionals would likely be the most reliable way to obtain information concerning depressive subtypes. Using only the BDI for this purpose is a limitation of the current study. Furthermore, ideally we would also have been able to assess diurnal variation and psychomotor symptoms of participants to include all the DSM‐IV criteria of melancholia. It has been shown that self‐reports of psychomotor retardation are not reliable.^32^ Considering that self‐reported questionnaires were used in this study, any information asked about psychomotor symptoms would likely have given very little reliable information. Diurnal variation was excluded due to it not being a part of the BDI‐IA that was used. The DSM does not require every criteria to be met in order to fulfill the diagnostic criteria. Therefore, we believe that the conclusions are valid even though one of the factors was not addressed.

There have been disagreements over whether the increased mortality of depression is simply reporting bias or a real phenomenon. One meta‐analysis shows that most studies found depression to be associated with higher mortality, but that many other studies found no such association.^33^ Another meta‐analysis did not find any increased mortality associated with depression.^5^ Even though our study was not able to show a large effect size we did find a significant difference in mortality between the melancholic depressive group and the non‐depressive group.

The participants in the current study were not eliminated from the participant pool based on any comorbidities. This means that a large number of the participants did have comorbidities that may have affected the mortality rates. However, because the participants were randomly selected the underlying assumption is that also the comorbidities would be representative of the general population. Removing participants based on comorbidities would have created a sample of much healthier participants, making the mortality calculations biased. Research has shown that certain comorbidities have a greater effect on mortality than others. Those with co‐morbid depression and anxiety had higher mortality risks,^30^ and those with diabetes and depression had a greater than additive effect of depression on mortality.^9^ It is also possible that diseases predisposing individuals to depression may cause an increased mortality rate.^29^ It would have been ideal to have data on the severity of disease for all diseases in the cohort. However, this information is not available. It is possible that different levels of somatic disease may have affected the depressive symptoms to some level. The fact that we have no way of controlling for this is a limitation of the study.

Depressed patients with a higher number of comorbidities have been shown to have the highest mortality risk, but they benefitted the most from depression intervention in terms of survival.^34^ Depression intervention has been shown to lower mortality risk in some groups of clinically depressed patients.^34^, ^35^ Research has shown that if the adverse effect of subthreshold depression and major depression on mortality were completely blocked, mortality could be reduced by up to 14%.^36^

Some research has focused on the differences between depressive episodes and remission. Depressive episodes can increase the risk of mortality for up to 20 years, but the risk decays if there is remission (Reference [37], pp. 1952–2011). Individuals whose depression is in remission have similar survival rates as non‐depressed patients.^29^ Data on the duration of the depressive episodes of the participants is not available. It may be possible that the duration may affect the effect size of the depression on factors such as mortality. Information on this could be a valuable addition to the study, and further our understanding of the effects of depressive subtypes. The lack of such information could be considered a limitation of the current study.

In the current study the SMR for the non‐depressive group were lower than for the general public. Likewise, another study also found a decreased SMR for the non‐depressive group.^28^ This may be explained by some survival bias where those with most comorbidities may have already passed away or did not participate in the study due to poor health. However, it is important to remember that this is the case for all study groups, and not just the non‐depressive group. Therefore, we did not only compare the groups to the general public, but we standardized the study sample's mortality per group based on the population of Finland, and then proceeded to compare the groups among themselves. This way we were able to compare the mortality to the population but most importantly compare the depressive groups. There is a possibility that any survival bias may not have been equally distributed between groups. Any data on this prior to our follow‐up does not exist and it is therefore impossible for us to make any claims regarding the distribution of any survival bias. If survival bias is unevenly distributed among the groups, it may cause the results to be skewed in any direction. It is a limitation of the current study that this information is not available.

Some strengths of this study are the length of the follow‐up period and the extensively phenotyped cohort. Furthermore, the large sample size and random selection of participants from the cohort are a strength. The homogenous age range has helped minimize the effect of age on mortality among the participants. In addition to these strengths, there are some limitations to the current study as well. One limitation is the relatively small size of the two depressive groups preventing comparisons by sex, which may have added value to the study. Furthermore, depression was not clinically diagnosed and followed‐up throughout the study, but rather self‐reported symptoms, which does not allow us to analyze for specific episodes and remissions. It is also likely that the participants exhibit some survival bias, making those who participated in the study healthier than members of the average population. Furthermore, genetics and the nature of the sample being of a very homogenous origin may result in the findings not necessarily being generalizable to other populations. The comorbidities of participants can also be viewed as a limitation since they are self‐reported but may have a substantial effect on mortality.

Further research comparing findings by both sex and depressive subtypes would be of interest. With a larger sample it may also be of value to compare causes of death between the depressive groups. Continuing to follow up within this same cohort and re‐evaluating the mortality causes and rates further down the line may also add value to what we know so far. It may also be of interest to look at current and remitted depression in the subtypes of depression as it has been shown that a current depressive episode, but not remission, was associated with a higher mortality risk.^11^

Factors that have been suggested to be at least part of the reason for the excess mortality are the cortisol and HPA‐axis dysregulation, lifestyle factors, and non‐compliance with treatment. Some other factors are cytokines and inflammatory marker dysregulation, lipid metabolism, and metabolic dysregulation. Furthermore, autonomic dysregulation has also been implicated. Of these causes melancholic depression is most closely associated with HPA axis hyperactivity, and non‐melancholic depression with inflammation and metabolic dysregulation.^12^, ^13^

Depression is a huge burden on both society and the individual, requiring better diagnosis and management. It can be difficult to detect due to its multifaceted presentation and differing symptomatology. Pathophysiological research on depression has moved forward but has yet to explain all aspects. However, experts recommend that specifying depressive subtypes should be performed more routinely.^38^

Depression is a heterogenous treatable disease that has been increasing in prevalence. Currently melancholic and non‐melancholic depression are distinguished by self‐reported symptoms rather than by established biomarkers. Up to half of individuals with depression are currently getting inadequate treatment for their condition.^39^ This tells us that there is a gap in our understanding of depression and its treatment. Better understanding of the pathophysiology of the depressive subtypes may be a factor in furthering both depression diagnosis and treatment.

The novelty of this study lies in it providing us with mortality information that differentiates between the depressive subtypes.

To conclude the melancholic depressive symptoms are associated with a higher overall and cumulative mortality. When compared to the non‐depressive, non‐melancholic and melancholic depressive symptoms both come with increased mortality on a population level.

## CONFLICT OF INTEREST

The authors declare no conflict of interest.

## Data Availability

The data that support the findings of this study are available from the corresponding author upon reasonable request.
